# Biphasic *Hoxd* Gene Expression in Shark Paired Fins Reveals an Ancient Origin of the Distal Limb Domain

**DOI:** 10.1371/journal.pone.0000754

**Published:** 2007-08-15

**Authors:** Renata Freitas, GuangJun Zhang, Martin J. Cohn

**Affiliations:** 1 Department of Zoology, Cancer/Genetics Research Complex, University of Florida, Gainesville, Florida, United Sates of America; 2 Department of Anatomy and Cell Biology, Cancer/Genetics Research Complex, University of Florida, Gainesville, Florida, United States of America; University of Maryland, United States of America

## Abstract

The evolutionary transition of fins to limbs involved development of a new suite of distal skeletal structures, the digits. During tetrapod limb development, genes at the 5′ end of the *HoxD* cluster are expressed in two spatiotemporally distinct phases. In the first phase, *Hoxd9-13* are activated sequentially and form nested domains along the anteroposterior axis of the limb. This initial phase patterns the limb from its proximal limit to the middle of the forearm. Later in development, a second wave of transcription results in 5′ *HoxD* gene expression along the distal end of the limb bud, which regulates formation of digits. Studies of zebrafish fins showed that the second phase of *Hox* expression does not occur, leading to the idea that the origin of digits was driven by addition of the distal Hox expression domain in the earliest tetrapods. Here we test this hypothesis by investigating *Hoxd* gene expression during paired fin development in the shark *Scyliorhinus canicula*, a member of the most basal lineage of jawed vertebrates. We report that at early stages, 5′*Hoxd* genes are expressed in anteroposteriorly nested patterns, consistent with the initial wave of *Hoxd* transcription in teleost and tetrapod paired appendages. Unexpectedly, a second phase of expression occurs at later stages of shark fin development, in which *Hoxd12* and *Hoxd13* are re-expressed along the distal margin of the fin buds. This second phase is similar to that observed in tetrapod limbs. The results indicate that a second, distal phase of *Hoxd* gene expression is not uniquely associated with tetrapod digit development, but is more likely a plesiomorphic condition present the common ancestor of chondrichthyans and osteichthyans. We propose that a temporal extension, rather than *de novo* activation, of *Hoxd* expression in the distal part of the fin may have led to the evolution of digits.

## Introduction

The origin of limbs was a defining event in the evolution of tetrapods. Important new discoveries in developmental genetics and vertebrate paleontology have enhanced our understanding of limb development and evolution [1]–[4]. The earliest fins appeared as median structures along the dorsal and ventral midlines in jawless fishes of the Lower Cambrian [5], [6]. These are likely homologs of the dorsal, anal and caudal fins of modern fishes. Median and paired fin development is controlled by a common set of molecular mechanisms [7]–[9]. Synthesis of paleontological and developmental data indicates that the genetic program for fin development originated in median fins, and the evolution of paired fins involved re-deployment of this genetic circuit to the lateral plate mesoderm [7]. The fin-to-limb transition occurred in the Late Devonian, when a new set of distal structures, the digits, appeared in lobed fins of stem-group tetrapods [10]. These early limbs were polydactylous, consisting of six (in *Tulerpeton*), seven (in *Ichthyostega*) and eight or more (in *Acanthostega*) short digits, with comparatively simple or poorly defined wrists and ankles [10]–[14].

Comparative developmental studies have demonstrated that the mechanisms controlling initiation, position, outgrowth and pattern are remarkably conserved between teleost fins and tetrapod limbs [4]. Fin buds and limb buds develop similarly at early stages; they emerge at discrete positions along body wall by localized maintenance of cell proliferation in lateral plate mesoderm [15], [16]. After initiation of budding, ectodermal cells along the distal edge of fin and limb buds undergo shape changes to form an apical ectodermal ridge (AER), which controls further outgrowth via secretion of fibroblast growth factors (Fgfs) into the underlying mesenchyme [17]–[20]. Both fins and limbs have a zone of polarizing activity (ZPA), a specialized population of mesenchymal cells at the posterior edge of the bud that controls anteroposterior patterning via secretion of the Sonic hedgehog (Shh) protein [21]–[23]. Fin and limb buds also exhibit a number of interesting differences at the cellular and the molecular levels. The AER is a transient structure in teleost fins; shortly after its appearance it elongates to form an apical ectodermal fold (AEF), within which dermal fin rays differentiate [15], [24]. This transition from a ridge to a fold has been proposed to account for the short endoskeletal and long dermal components of teleost fins, based on evidence that elimination of the ridge in chick embryos leads to an arrest of endoskeletal development in the underlying mesenchyme [25].

*Hoxd* genes regulate the anteroposterior pattern of both fins and limbs by establishing an early map of cell identity that is important for specification of the ZPA [26]. *Hoxd* genes are expressed in highly dynamic patterns during limb development. Early work suggested that there are three phases of Hox expression in tetrapod limbs [27], but it is now clear that the original phases I and II are unified mechanistically during a single early wave of transcriptional activity that is now considered phase I [28]. Prior to the onset of limb budding, the most anterior gene, *Hoxd9*, is expressed in lateral plate mesoderm up to the pectoral level [29]. As limb budding commences, *Hoxd9* expression is maintained and the neighboring *Hoxd10-Hoxd13* genes are activated sequentially. This produces a spatially and temporally collinear pattern of nested expression domains along the anteroposterior axis of fins and limbs, with the *Hoxd13* domain being the most posteriorly restricted [27], [30], [31]. A similar pattern is established in the early pelvic fin/limb bud. In tetrapods, a second wave of transcriptional activity results in 5′ *Hoxd* genes being re-expressed along the distal margin of the limb buds, in the area of the prospective digits [32]. During this second phase, *Hoxd13* is expressed in all of the developing digits whereas *Hoxd12* and *Hoxd11* are expressed in all but the anteriormost digit. By contrast, this late phase of *Hox* expression was not observed during zebrafish fin development [31], [33]. These differences between zebrafish and tetrapods were interpreted in light of the functional requirement of *Hoxd* genes for digit development and the emerging picture of early tetrapod digit evolution, and an elegant new hypothesis proposed that digits are neomorphic structures that resulted from acquisition of the late distal domain of *Hoxd* gene expression during tetrapod evolution [31], [33], [34].

Genetic analyses of *HoxD* gene regulation in mice have shown that the two phases of expression within the limb buds result from two independent waves of transcriptional activation. The first wave involves the action of opposite regulatory modules located outside of the cluster, which leads to sequential transcription of *HoxD* genes from the 3′ to the 5′ end of the complex [28]. The second wave of transcription is regulated by two enhancer-containing domains, the Global Control Region (GCR) and the Prox region, which are situated centromeric to the cluster and govern the re-expression of 5′ *HoxD* genes in the distal region of the limb [35], [36]. The independent regulation of the first and second waves of *HoxD* gene expression during mouse limb development is consistent with the proposal that proximal and distal parts of the limb have distinct evolutionary histories [28]. With respect to the evolutionary origin of digits, these data suggested that a novel enhancer sequences emerged outside the *Hoxd* cluster and resulted in distal activation of *Hoxd* expression, or that the preexisting regulatory modules were co-opted to perform this function during the transition from fins to limbs [28]. Both of these scenarios operate under the assumption that the second wave of *Hoxd* expression in the distal aspect of the limb is unique to tetrapods and contributed to the evolutionary origin of digits.

Here we investigate whether the monophasic expression of *Hoxd* genes observed in zebrafish fin buds is representative of the primitive condition for gnathostome (jawed vertebrate) fins. Zebrafish fin morphology is highly derived relative to other actinopterygians, sarcopterygians and chondrichthyans. A tribasal fin skeleton, containing a propterygium anteriorly, a mesopterygium in the middle and a metapterygium posteriorly, is widely considered to be the primitive pattern for gnathostomes [37] (for a detailed discussion of mesopterygial evolution, see ref [38]). Among crown-group vertebrates, all three elements are found in most chondrichthyans, and basal actinopterygians show clear homologs of the propterygium and metapterygium, with the mesopterygium represented by a varied number of middle proximal radials [37]–[40]. In teleost fishes, the metapterygium has been lost and the remaining radials are reduced [15], [37], [40], [41]. In addition, teleosts have undergone an additional round of genome duplication, which has provided them with seven Hox clusters [42]. By contrast, chondrichthyans are the most basal lineage of extant gnathostomes, and shark fins retain many plesiomorphic features, including a tribasal skeleton from which an elaborate series of radials project distally [1], [43]. In addition, sharks have been reported to possess four Hox clusters orthologous to those of non-teleostean gnathostomes, including coelacanths, birds and mammals [7], [44]–[46]. Thus, chondrichthyans provide a unique opportunity to investigate paired fin development in the sister group to the bony fishes, which could shed light on the mechanisms that operated during early evolution of paired fins.

In this report, we first examine skeletal development in the fins of the catshark (*Scyliorhinus canicula*), and find, at both cellular and molecular levels, striking similarities to tetrapod patterns of skeletogenesis as well as differences relative to the zebrafish pattern. In order to identify the primitive role of 5′*Hoxd* genes in fin evolution, we analyze the expression pattern of these genes during catshark paired fin development. At early stages of fin development, 5′*Hoxd* genes are expressed in collinear, nested patterns along the anteroposterior axis of the fins, which resemble the initial wave of *Hoxd* transcription that occurs in the paired appendages of other gnathostomes. We also describe an unexpected second wave of expression at later stages of shark fin development, in which *Hoxd12* and *Hoxd13* are re-expressed along the distal margin of the paired fin buds. The results indicate that biphasic, distal expression of *Hoxd* genes is not uniquely associated with tetrapod digit development, but is more likely a plesiomorphic condition that was present the common ancestor of chondrichthyans and osteichthyans.

## Results

### Skeletal development in shark paired fins

**Figure 1:**
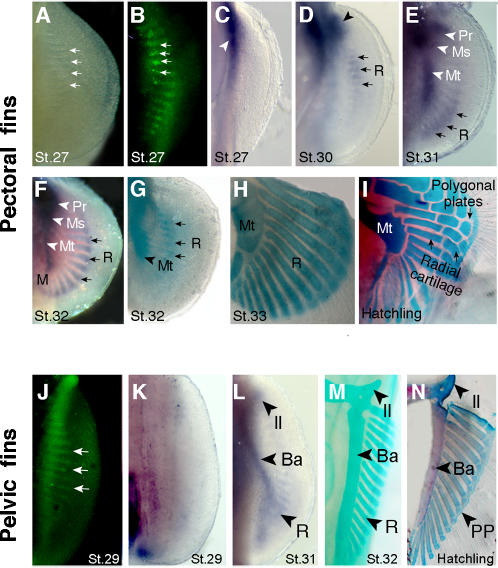
Endoskeletal development in catshark pectoral and pelvic fins. Ventral views of pectoral (A–I) and pelvic (J–N) fins. Stages (St.) of development indicated at bottom of each panel. (A) Light micrograph of pectoral fin showing gaps in the pectoral fin plate. (B) Acridine orange staining (green fluorescence) shows apoptotic cells in the gaps observed in panel A. Arrows in A and B mark four examples. (C) *Sox8* expression marks initiation of chondrogenesis in the pectoral girdle region (arrowhead). Note absence of chondrogenesis in the fin plate at this stage. (D) *Sox8* expression marks initiation of chondrogenesis in anterior part of the fin plate, in basal cartilages (arrowhead) and radials (arrows). (E) *Sox8* domain prefigures development of the basal cartilages along the anteroposterior axis of the fin: Pr, propterygium; Ms, mesopterygium; Mt, metapterygium; R, radials. Arrows mark expression in the most posterior radials. (F) *Sox8* expression in basal cartilages (arrowheads) and in all radials along the anteroposterior axis (subset of radials marked with arrows). (G, H) Alcian green staining of pectoral fins. Note that radials chondrify in domains pre-established by *Sox8* expression domains (compare with panels F and G). Chondrified, unsegmented radials are seen in H. (I) Alcian blue and alizarin red stained pectoral fin showing a fully developed cartilaginous endoskeleton at the time of hatching. Note segmentation of proximal radials, intermediate radials and distal polygonal plates (compare panels H and I). (J) Acridine orange-positive cells in gaps of the pelvic fin plate. (K) *Sox8* expression marks initiation of chondrogenesis proximal, posterior region of fin. Note absence of chondrogenesis in the fin plate at this stage. (L) *Sox8* expression prefigures development of endoskeletal elements in the pelvic fin. Il, iliac process; Ba, basipterygium; R, radials. (M) Alcian green staining of the pelvic fin showing chondrified unsegmented radials. (N) Alcian blue and alizarin red staining of the pelvic fin showing fully developed cartilaginous endoskeleton at hatching. Note segmentation of the radials into distal polygonal plates (PP) and proximal radials (compare panels M and N).

To identify how individual cartilage elements form in catshark fins, we asked whether the fin endoskeleton develops from a cartilagenous plate that perforates to form the individual radials, as in teleosts [15], or by formation of individual cartilage condensations, as in tetrapods [47]. Catshark pectoral fins buds consist of dense mesenchyme at stage 27, and we observed serially-spaced gaps in the proximal region of the buds (Fig. 1A). To determine whether these discontinuities result from apoptosis, we stained live embryos with acridine orange, a vital stain that has been shown to selectively label apoptotic cells [48]. Proximodistally-oriented stripes of acridine orange-positive cells were observed in the inter-radial spaces (Fig. 1B), indicating that these spaces correspond to localized domains of apoptosis. To determine the temporal relationship of this segmentation process to chondrogenic differentiation, we analyzed the expression of *Sox8*, a SRY-related gene that marks chondrogenic cells before they are detectable by alcian blue staining [49]. At stage 27, *Sox8* expression was restricted to the proximal-anterior aspect of the fin, in the region of the prospective pectoral girdle (Fig. 1C). The *Sox8* domain then spread distally and posteriorly, revealing the beginning of chondrification of the basal cartilages (Fig. 1D). Simultaneously, stripes of expression were detected in the fin plate, at the sites the anterior radials (Fig. 1D). Between stages 31 and 32, *Sox8* expression expanded posteriorly and the expression domains marked the positions of the three basal cartilages (metapterygium, mesopterygium and propterygium) and the associated radials (Fig. 1E, F). Comparison of *Sox8* expression with alcian blue staining at stage 32 showed that the radials chondrified in domains pre-established by the expression of *Sox8* (compare Figs. 1F and 1G). Thus, perforation of fin bud mesenchyme occurs by apoptosis before the onset of chondrogenic differentiation. Prior to hatching, the radial cartilages increased in size and further segmented to form two segments per radial and ending in distal polygonal plates (compare Figs. 1H and 1I).

We next investigated whether the pelvic fins skeleton develops by the same mechanisms. In stage 29 pelvic fin buds, proximodistally-oriented stripes of apoptotic cells were detected along the anteroposterior axis (Fig. 1J). Analysis of *Sox8* expression at the same stage revealed a strong proximal-posterior expression domain and weaker expression proximally and anteriorly (Fig. 1K). By stage 31, the *Sox8* expression pattern prefigured the entire endoskeleton, including the pelvic basal cartilage (basipterygium) and the adjacent radials (Fig. 1L). The pelvic fin radials continued to increase size and underwent further segmentation to form the distal polygonal plates (Fig. 1M, N). The results indicate that undifferentiated pelvic mesenchyme is sculpted by apoptosis, and proximal-to-distal chondrification gives rise to the basal elements and radials, as in pectoral fins. Chondrification of the pectoral and pelvic fin skeletons in catsharks is therefore more similar to patterns reported for tetrapod limbs than for teleost fins.

### *Hoxd* gene expression during shark pectoral fin development

**Figure 2:**
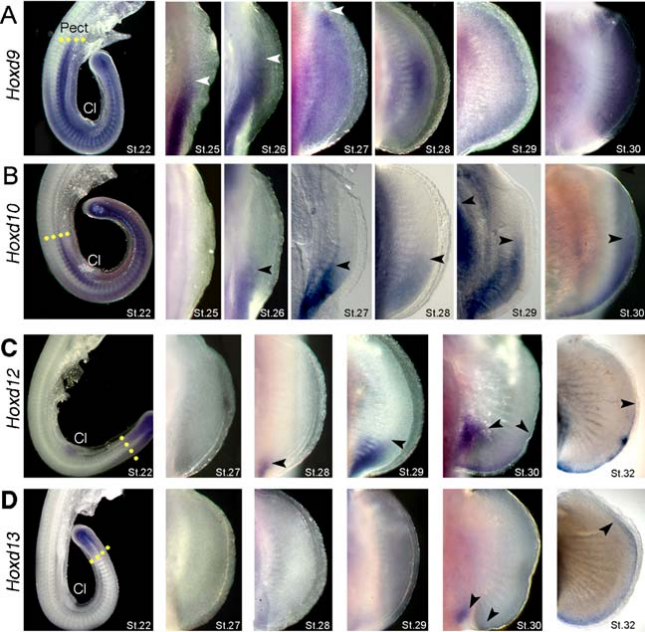
Expression of *Hoxd* genes in catshark pectoral fins. Stages of development indicated in lower right corners of each panel. (A–D) Whole mount *in situ* hybridizations showing expression of *Hoxd9* (A), *Hoxd10* (B), *Hoxd12* (C) and *Hoxd13* (D). Pect, Pectoral fin bud; Cl, cloaca. Note anterior expansion of *Hoxd12* and *Hoxd13* in distal fin at stage 32. Arrows mark anterior limits of expression. Yellow dotted lines in the left column mark the anterior boundaries of expression at stage 22.

**Figure 3:**
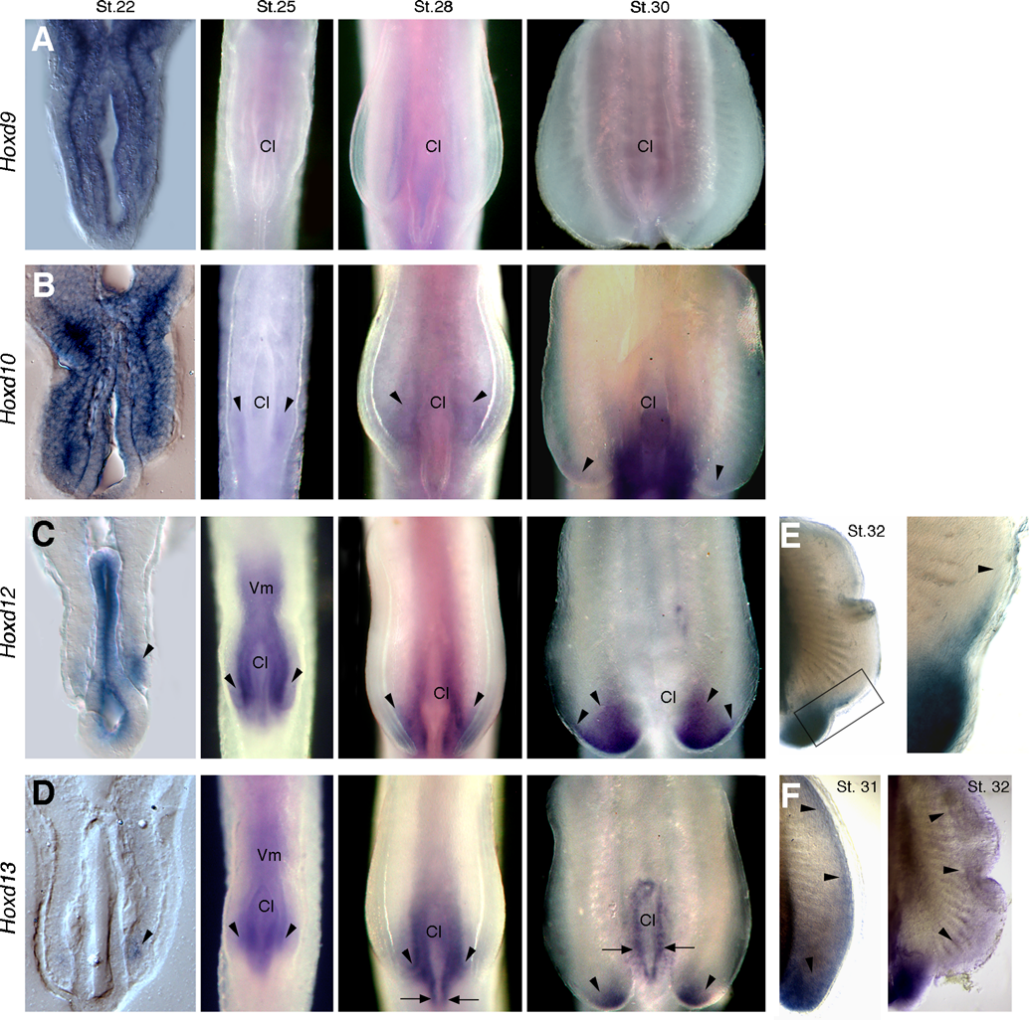
Expression of *Hoxd* genes in catshark pelvic fins. Stages of development indicated in the top of each column in A–D and in upper right corner in E and F. Left column shows transverse histological sections at level of cloaca (Cl) and pelvic fins. All other panels show whole mounts in ventral view. (A–D) Whole mount *in situ* hybridizations showing expression of *Hoxd9* (A), *Hoxd10* (B), *Hoxd12* (C) and *Hoxd13* (D). Arrowheads mark expression in pelvic fin buds. Arrows in D mark expression in cloacal epithelium. (E, F) Pelvic fins showing expression of *Hoxd12* at stage 32 (E) and *Hoxd13* at stages 31 and 32 (F). Boxed area in E is shown in high magnification at right. Arrowheads in E mark anterior limits of expression, and in F they outline the extent of the distal *Hoxd13* domain.

In light of the primitive morphological characters present in shark pectoral fins [43], and our finding that development of the fin skeleton in shark embryos is strikingly different to that of zebrafish, we reasoned that the dynamics of *Hoxd* gene expression in shark paired fins may provide insights into the patterns that operated in the common ancestor of chondrichthyans and osteichthyans. We therefore examined *Hoxd9-13* expression during development of catshark pectoral and pelvic appendages. At early stages of pectoral fin budding, *Hoxd* genes were expressed in collinear, nested domains along the trunk, with the most anteriorly-expressed gene, *Hoxd9,* marking the posterior limit of the emerging pectoral fins (Fig. 2A, stage 22). *Hoxd10* extended up to the level of the mid-flank, between the pectoral and the pelvic fin regions (Fig. 2B, stage 22). Both genes were expressed in the region of the prospective pelvic fins, on either side of the cloacal region (Fig. 2A, B and Fig. 3A,B, stage 22). At the same stage, *Hoxd12* was detected in the tail bud and cloacal regions (Fig. 2C and Fig. 3C), and *Hoxd13* was expressed further posteriorly in both of these domains (Fig. 2D and Fig. 3D). As the pectoral fin buds became dorsoventrally flattened, *Hoxd9* expression extended anteriorly throughout the fin mesenchyme, terminating at the anterior margin of the fin by stage 27 (Fig. 2A). *Hoxd10* was detectable in the pectoral fins beginning at stage 26 (Fig. 2B). The *Hoxd10* expression domain continued to spread anteriorly, however its anterior limit remained posterior to that of *Hoxd9* (compare Fig. 2A with 2B). *Hoxd12* expression appeared in the posterior region of the pectoral fin bud between stages 27 and 28, and encompassed the posterior radials at stage 29 (Fig. 2C). *Hoxd13* transcripts were not detectable in the pectoral fin bud before stage 29 (Fig. 2D). The results show that during early development of catshark pectoral fins, *Hoxd* genes are activated in a spatially and temporally collinear pattern that resembles the first phase of *Hoxd* expression in tetrapod limbs and teleost fins.

In order to determine whether the monophasic expression pattern reported in zebrafish [31] is plesiomorphic for gnathostomes, we went on to examine *Hoxd* expression at later stages of catshark fin development. Between stages 28 and 30, *Hoxd9* expression became restricted to the distal mesenchyme of the fin (Fig. 2A). At stage 29, *Hoxd10* was separated into proximal and distal domains, and by stage 30 *Hoxd10* expression was restricted to the distal edge of the pectoral fin (Fig. 2B). *Hoxd12* also exhibited two separate domains of expression at stage 30; a proximal domain encompassed the posterior radials and a distal domain was observed along beneath the distal ectoderm along the posterior 1/3 of the fin (Fig. 2C). The distal domain continued to spread anteriorly along the distal edge of the fin, covering more than half of the distal margin by stage 32 (Fig. 2C). *Hoxd13* was first detected in the posterior-proximal fin bud at stage 30 (Fig. 2D). *Hoxd13* expression also shifted distally and anteriorly, forming an elongated, narrow domain that extended approximately 2/3 of the way along the distal-most mesenchyme of the pectoral fin at stage 32 (Fig. 2D). Both the anterior-distal expansion, in which *Hoxd13* extended anterior to *Hoxd12*, and the proximal-distal subdivision of the *Hoxd10* and *Hoxd12* domains, resembled the second/late phase of *Hoxd* gene expression reported for tetrapod limbs [27]. A noteworthy difference, however, is the distal fin domain in sharks is extremely narrow relative to the distal limb domain in tetrapods.

### *Hoxd* gene expression during shark pelvic fin development

**Figure 4:**
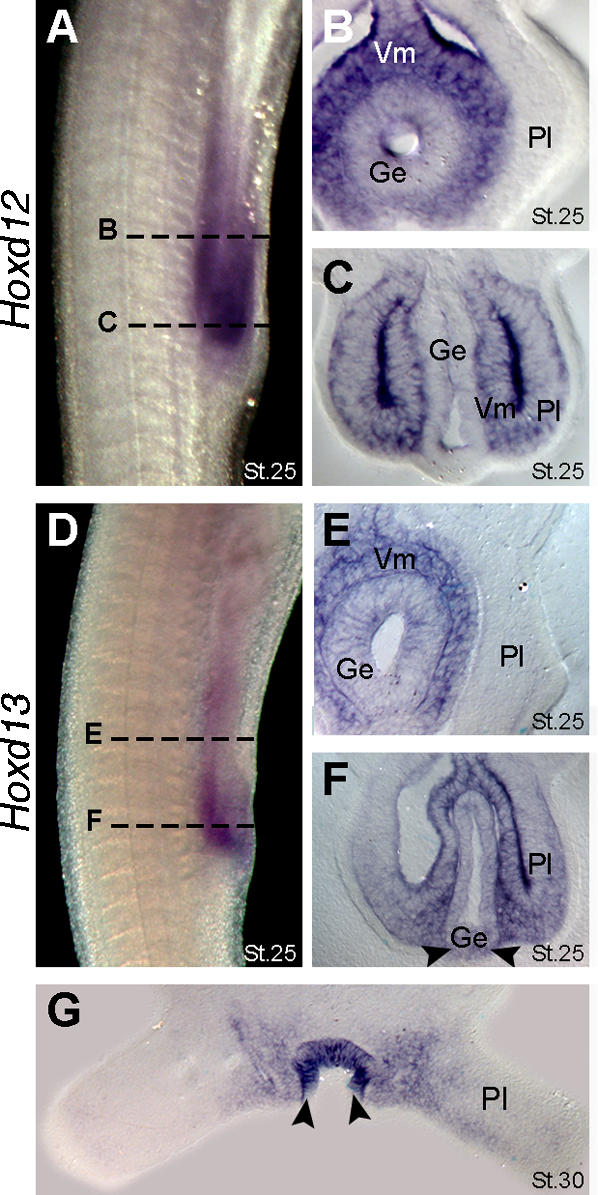
Expression of *Hoxd12* and *Hoxd13* in the cloacal region of catsharks. (A) Lateral view of pelvic fin region showing *Hoxd12* expression at stage 25. Dashed lines mark the approximate planes of section showed in panels B and C. (B) Transverse section showing *Hoxd12* expression in visceral mesoderm (Vm) and gut endoderm (Ge). Note absence of *Hoxd12* expression in anterior part of the pelvic fin (Pl). (C) Transverse section showing *Hoxd12* expression in the posterior part of pelvic fin and adjacent visceral mesoderm. Note absence of *Hoxd12* expression in the gut endoderm. (D) Lateral view of the pelvic fin region showing *Hoxd13* expression at stage 25. Note that *Hoxd13* domain lies posterior to *Hoxd12* domain (compare with panel A). Dashed lines mark the approximate planes of the section showed in panels E and F. (E) Transverse section showing *Hoxd13* expression in the visceral mesoderm and gut endoderm. Note absence of *Hoxd13* expression in the anterior part of pelvic fin. (F) Transverse section showing *Hoxd13* expression in the posterior part of the fin, visceral mesoderm and ventral endoderm. Arrowheads mark expression in endoderm (contrast with absence of *Hoxd12* in endoderm in panel C). (G) Transverse section throughout the pelvic fins at stage 30 showing *Hoxd13* expression in the cloacal epithelium (arrowheads).

Initiation of pelvic fin budding was preceded by the expression of *Hoxd9* in the somatic layer of the lateral plate mesoderm at the cloacal level (Fig. 3A, stage 22). By the time pelvic fins emerged, however, *Hoxd9* was no longer detectable in the fin mesenchyme (Fig. 3A, stage 25). Similarly, *Hoxd10* was first expressed throughout the region of the prospective pelvic fins (Fig. 3B, stage 22), but by the onset of budding, *Hoxd10* had become restricted to the posterior mesenchyme (Fig. 3B, stage 25). *Hoxd12* was expressed in posterior mesenchyme of the pelvic fins from the initial stages of outgrowth (Fig. 3C and Fig. 4A–C). *Hoxd13* was expressed even further posteriorly in pelvic appendages (Fig. 3D and Fig. 4D–F). *Hoxd12* and *Hoxd13* were maintained in the posterior regions of the pelvic fins, in the swellings from which the male claspers develop (Fig. 3C and 3D, stage 30). Both *Hoxd12* and *Hoxd13* then exhibited a second phase of expression, in domains that extended anteriorly as narrow bands of expression along the distal most mesenchyme of the pelvic fin buds (Fig. 3E, F). At stage 31, a new distal domain of *Hoxd13* could be observed extending along the entire distal margin of the pelvic fin, and expression persisted throughout the distal-most mesenchyme at stage 32 (Fig. 3F). By stage 32, a narrow band of *Hoxd12* expression also extended anterior to the clasper, immediately under the distal ectoderm (Fig. 3E). Thus, in the developing pectoral and pelvic fins of the catshark, *5*′*Hoxd* genes are expressed in dynamic, biphasic patterns, and the second phase shows a reversal of temporal and spatial collinearity similar to that found in shark pectoral fins and in tetrapod limbs.

Interestingly, *Hoxd12* and *Hoxd13* also displayed distinct boundaries of expression in the hindgut. *Hoxd13* was expressed further posteriorly than *Hoxd12* in splanchnic (visceral) mesoderm and endoderm at the cloacal level between stages 25 and 28 (Fig. 3C, D and Fig. 4). By stage 30, *Hoxd12* expression had been downregulated in the cloacal region (Fig. 3C) and *Hoxd13* transcripts became restricted to the cloacal epithelium (Fig. 3D and Fig. 4G). Taken together, the results show that the biphasic expression of 5′ Hoxd genes during paired appendage development and their expression in the anogenital region are conserved to the most basal lineage of crown-group gnathostomes.

## Discussion

During tetrapod limb development, two phases of *Hoxd* gene expression result from two distinct waves of transcriptional activity, with the first wave controlling pattering of the limb up to the forearm, and the second wave regulating formation of the digits [28], [50], [51]. Previous analyses of *Hox* gene expression patterns in tetrapod limbs and zebrafish fins showed that zebrafish fins exhibit only the first phase of expression, in which the genes are activated in anteroposteriorly nested domains [31], [52]. The absence of a second phase of *Hoxd* gene expression in zebrafish fins, along with functional studies showing the requirement of these genes for digit development in tetrapods, led to the idea that acquisition of a novel, second phase of *Hoxd* gene expression facilitated the origin of tetrapod digits. Our discovery of two phases of *Hoxd* expression during pectoral and pelvic fin development in a chondrichthyan raises the possibility that biphasic expression evolved before the divergence of chondrichthyans and osteichthyans.

### Biphasic *Hoxd* gene expression in shark fins

#### Phase I

We found that the early patterns of *Hoxd* gene expression in sharks are similar to those reported for zebrafish and for a variety of tetrapods [27], [30], [31], [52]–[54]. During this first phase, *Hoxd* genes are activated sequentially in shark lateral plate mesoderm and they are expressed in spatially collinear patterns. The dynamics of *Hoxd9* expression during early development of shark pectoral fins closely resemble the pattern observed during chick limb initiation, in which *Hoxd9* is expressed initially up to the boundary between the flank and prospective forelimb bud, and the boundary then shifts anteriorly to be expressed throughout the forelimb bud [29]. Previous investigators have recognized the apparent discontinuity between the *Hoxd* gene expression domains in the forelimb relative to the trunk, and numerous models have been proposed to explain how *Hoxd13* came to be expressed at such an anterior position during vertebrate evolution [55]–[58]. The results reported here, together with earlier work in the chick and mouse [29], [59], suggest that this can be explained by sequential activation of the *Hoxd* complex in lateral plate mesodermal cells that expressed *Hoxd9* at early stages of limb initiation. Thus, *Hoxd13* expression in the forelimb and pectoral fin is simply the end result of collinear transcription in lateral plate mesoderm cells that undergo sustained proliferation, probably under the control of the signaling molecules produced within the limb bud.

The early polarity of *Hoxd* expression in mouse limbs establishes the anteroposterior asymmetry of the endoskeleton elements [26], [60]. This is achieved by polarized activation of *Shh* at the posterior end of the limb bud. In turn, maintenance of these collinear patterns is controlled, at least in part, by Shh [26]. We found similar patterns of expression during catshark fin development, however the last gene in the complex, *Hoxd13*, is activated at a relatively late stage of fin development. Interestingly, recent work has shown that *Shh* also is activated at a late stage of chondrichthyan fin development [61]. If posterior *Hoxd* expression is required for transcription of *Shh* in the posterior part of fins and limbs, then the slow activation of the *Hoxd* complex in chondrichthyan fin development may account for the delay in *Shh* expression.

#### Phase II

More surprising is the discovery that the initial phase of collinear *Hoxd* gene expression is followed by a second phase, in which *Hoxd12* and *Hoxd13* are activated at the distal end of the fin bud. This anterior-distal expression of *Hoxd* genes has been proposed to be a key character of the tetrapod limb that distinguishes limbs from fins [62]. Moreover, the requirement of this distal *Hox* gene expression for tetrapod digit development, along with the finding that this phase of transcription is controlled by separate regulatory modules, has led to the widely-held view that the origin of digits was driven by acquisition of a novel, late phase of Hox gene expression [31], [62]. The hypothesis involves an assumption that the pattern observed in zebrafish is representative of the primitive condition, which reveals a limitation of two-taxa comparisons [38], [41]. Our results suggest two possible explanations for the reported difference of the zebrafish pattern; either the single phase of *Hoxd* gene expression reported for zebrafish is a derived state, or a second phase of expression occurs at stages later than (or involves genes different to) those examined in previous reports. Our analysis of skeletal development in shark fins shows a process with greater similarity to the tetrapod limb than to the teleost fin. The latter undergoes differentiation of the fin bud mesenchyme into a chondrogenic plate, which it then segments to form the individual bones of the fin, whereas shark and tetrapod appendicular skeletons develop by polarized condensation of separate prechondrogenic elements that then differentiate into cartilage. The teleost pectoral fin skeleton is also stunted relative to the elaborate distal endoskeleton of sharks and basal actinopterygians [38], [39], [41], which suggests that failure of zebrafish fin buds to execute the second phase of *Hoxd* gene expression may underlie the developmental truncation of their fin skeletons. Our results indicate that biphasic, distal expression of *Hoxd* genes is not uniquely associated with tetrapod digit development, but is more likely a plesiomorphic condition for gnathostomes.

### Patterning of the distal appendicular skeleton

Another striking similarity between the shark and tetrapod patterns of *Hoxd* gene expression is that proximal and distal domains are separated by a zone of non-expressing cells at late stages of development, and late expression appears to be regionalized along the proximodistal axis. In tetrapod limbs, the appearance of collinear *Hoxd* expression along the proximodistal axis of the limb has been termed “virtual collinearity”, which arises as an artifact of the two independently-regulated waves of collinear activation, the early/proximal phase controlled by the ELCR and the late/distal phase controlled by GCR/Prox [28], [35]. Our findings that shark fins exhibit distinct early/proximal and late/distal *Hoxd* expression domains, which later appear proximodistally subdivided, suggests that the proximal and distal limb may have been under modular developmental control from an early point in gnathostome fin evolution. This also raises the possibility that factors from the AER may be involved in maintaining expression at the distal tip of the fin bud (perhaps by keeping these cells in a proliferative state). It is therefore interesting that the shark AER expresses Fgf8 [7] , a factor known to mediate this function in tetrapods [17], [63]–[65].

Sharks develop paired fins as localized outgrowths of the lateral plate mesoderm at discrete positions along the body axis, and these fin buds then develop an AER that later becomes an AEF [66]–[68]. This is similar to fin budding in bony fishes [15]. The first phase of endoskeletal development superficially resembles that which occurs in bony fishes; proximally, fin bud mesenchyme condenses and localized apoptosis generates perforations of the fin plate [66]. However, our *Sox8* data demonstrate that catshark fin bud mesenchyme does not undergo chondrogenic differentiation prior to the condensation of individual radials, which contrasts with patterns described for actinopterygians and some species of shark, which undergo early formation of a chondrogenic plate that later perforates to separate the radials [15]. Catshark radials differentiate as individual elements, which is similar to the skeletogenic process in tetrapods limbs [47]. The dynamics of *Sox8* expression also revealed that chondrogenesis in catshark pectoral fins follows an anterior to posterior progression, starting in the prospective pectoral girdle. Similar directionality occurs in urodele amphibian limbs, whereas in amniotes the polarity of chondrogenesis generally is from posterior to anterior [69]. Our finding that the second phase of *Hoxd* expression occurs distal to the region of differentiated cartilage is consistent with idea that the second phase governs cell proliferation in the distal limb bud [38], [69].

### Relationship of *Hoxd* expression to genital development

During development of the shark gut, 5′ *Hoxd* genes are expressed in cloacal mesoderm and endoderm. Similar patterns were observed in zebrafish [33]. In mammals, *Hoxd13* is required for anorectal and external genital development, and its expression in the genital tubercle and digits is under shared genomic regulation [51], [70]. Co-regulation of *Hoxd* gene expression in these tissues led to the hypothesis that the evolution of terapod digits and external genitalia may have been coordinated by a shared mechanism. Our results suggest a more ancient origin for *Hoxd* expression in the distal aspect of the fin buds and in the cloaca. Interestingly, *Shh*, which is expressed in the cloaca-derived urethral plate of the mouse genital tubercle and is required for outgrowth of the phallus [71], [72], is also expressed in cloacal endoderm of chondricthyans (our unpublished data) and teleosts [73]. If genes required for external genital development were expressed in the cloaca before the evolution of a phallus, then sustained exposure of these cell populations to a proliferative cue may have been sufficient for development of a patterned genital organ.

### The origin of digits

This study allows reconsideration of the idea that the distal expression of *Hoxd* genes was associated with the origin of digits. Based on evidence that the second wave of transcriptional activity in the mouse autopod is controlled by its own regulatory modules and is required for digit development, and that this phase is absent in zebrafish (which lack digits), this domain of expression has been considered a character of the autopod. It is therefore tempting to speculate that the distal domain of *Hoxd* expression in sharks may define a population of cells with an autopodial identity, as was suggested recently for paddlefish [74], however caution should be exercised in making inferences concerning homology based on gene expression data. Rather than considering this distal domain of expression to be digit-like, we suggest that distal domain can be interpreted as a reflection of distal positional identity at a cellular (not anatomical) level. Thus, the data do not indicate structural homology of distal elements in fins and limbs, but instead suggest that cells at the tips of fins and limbs may be responding to similar positional cues. This interpretation is consistent with the proposal that, in all animal appendages, *Hox* genes function to specify two developmental modules, proximal and distal, and these modules are not linked to specific anatomical landmarks [75].

**Figure 5:**
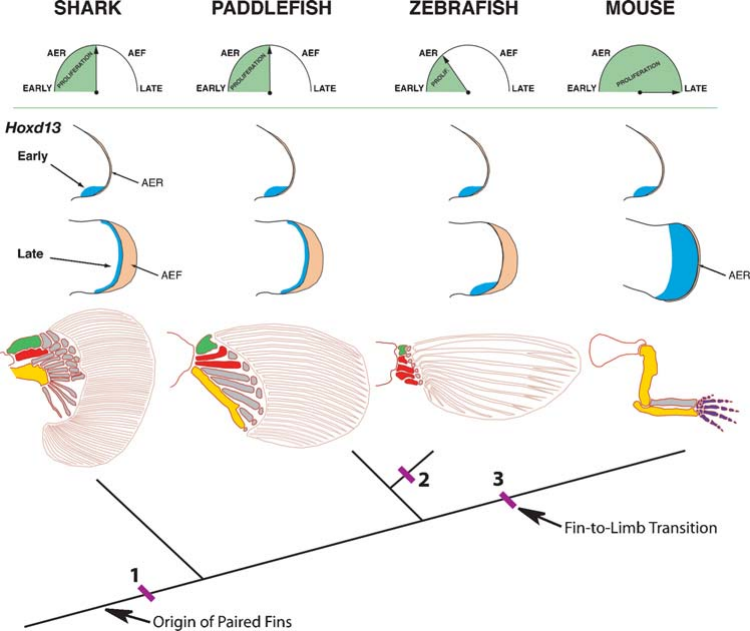
Model for the origin of digits by temporal extension of distal *Hoxd13* expression. Tree shows phylogenetic relationships of shark, paddlefish, zebrafish and mouse. Top row shows hypothetical timing for the transition of the apical ectodermal ridge (AER) to apical ectodermal fold (AEF); note that the mouse maintains an AER and does not form an AEF. Green shading represents proliferative period for endoskeletal progenitor cells. Middle rows show *Hoxd13* expression domains (blue) at early and late stages of fin and limb bud outgrowth. AER and AEF are shaded orange. Bottom row shows pectoral appendicular skeleton for each taxon. Endoskeletal bones are shaded as follows: green, propterygium; red, mesopterygium, yellow, metapterygium. Dermal fin rays are shown as unshaded elements within fin blade. The model suggests that a second phase of distal *Hoxd13* expression was present in the paired fins of the common ancestor of chondrichthyans and osteichthyans (at position 1), and that loss of the distal *Hoxd13* domain in teleosts (position 2) and its spatial expansion in tetrapods (position 3) may have been associated with temporal modulation of endoskeletal progenitor cell proliferation. Early conversion of the AER to an AEF would be expected to truncate or eliminate phase II expression of *Hoxd13* and reduce the fin endoskeleton, as seen in zebrafish, whereas prolonged signaling by the AER would be expected to extend Phase II and expand the *Hoxd13* domain, giving rise to digits in the tetrapod lineage. Clock model after [25]; skeletal patterns after [15], [31], [40], [74].

What, then, do these data tell us about the origin of digits? Firstly, the discovery that the second wave of *Hoxd* gene expression at the distal tip of paired appendages can be extended to the chondrichthyan lineage allows us to exclude the hypothesis that a novel domain of distal *Hoxd* expression first appeared in stem-group tetrapods. Secondly, distal *Hoxd* expression does not itself lead to development of an autopod. The third point relates to the demonstration by Duboule and co-workers that 5′ *HoxD* and *HoxA* genes are required for proliferation of skeletogenic precursors cells in the limb [32], [76]. The distal *Hoxd* domain in shark fins may regulate cell proliferation beneath the AER. As such, its presence at late stages of shark fin and tetrapod limb development, and its absence from zebrafish, would fit with elaboration of the distal skeleton in the former and its truncation in the latter. It is therefore intriguing that the size of the distal expression domain in sharks is extremely narrow relative to that of tetrapods. The pivotal event with respect to the origin of digits may have been a temporal extension of the second transcriptional wave, which would have led to a sustained period of cell proliferation, thereby increasing the size of the distal *Hoxd* domain, at the terminus of the limb (Fig. 5).

Whether expansion of the distal *Hoxd* domain at the fin-to-limb transition was accomplished by modulation of existing regulatory elements, evolution of new enhancer sequences, or by sustained production of mitogenic factors, such as Fgfs from the apical ridge, is unknown. Expansion of the primitive distal *Hoxd* domain by sustained signaling from the AER is consistent with Thorogood's proposal that the extent of endoskeletal development in actinopterygian and sarcopterygian appendages is controlled by the timing of the transition of the AER to an AEF [25]. According to the model, delaying this switch would result in an extended period of AER signaling activity and, in turn, produce a more elaborate endoskeleton. Our findings may provide a molecular mechanism for Thorogood's model (Fig. 5). Given that *Hoxd13* expression in the limb bud is maintained by Fgfs from the AER [17], [35], an attractive possibility is that delayed conversion of the AER to an AEF could have prolonged the period of Fgf signaling, which would result in sustained *Hoxd13* expression in the distal part of the fin. A consequence of this delay would be a spatial expansion of the distal *Hoxd13* expression domain and an associated increase in cell proliferation, both of which would be required for elaboration of the distal limb skeleton (Fig. 5). Thus, a temporal extension, rather than *de novo* activation, of *Hoxd13* expression in the distal part of the fin may have contributed to development of digits during the evolutionary transition of fins to limbs.

## Materials and Methods

### Collection and staging of embryos

*Scyliorhinus canicula* eggs were collected from Menai Strait (North Wales). Embryos were isolated from the eggshells, dissected from the yolk sac in ice-cold phosphate buffered saline solution (PBS) and staged according to Ballard et al [67]

### Whole-mount cartilage staining

For alcian green staining, embryos were washed in PBS, fixed overnight in 5% trichloroacetic acid (TCA) and transferred to 0.1% alcian green in acid ethanol. Stained specimens were differentiated in acid ethanol, dehydrated in ethanol and cleared in benzyl alcohol:benzyl benzoate (BABB). Hatchling specimens were fixed in 80% ethanol and eviscerated before being stained with alcian blue and alizarin red as described previously [77].

### Acridine orange staining

Acridine orange (AO) was used to identify apoptotic cells, following the method of Abrams et al [48]. Embryos were rinsed briefly in PBS, after being separated from the yolk sac, and incubated in 0.5 µg/ml AO in PBS at 37°C for 30 minutes in the dark. Specimens were then examined and photographed under UV fluorescence.

### Whole mount *in situ* hybridization

Fragments of *5′Hoxd* genes and *Sox8* were used to generate digoxigenin-labelled riboprobes as described previously [7], [78]. *In situ* hybridization of catshark embryos were carried out using our published modification [78] to the method of Nieto et al [79], and a treatment with 60 µg/ml proteinase K was performed on embryos at stages 32 and 33. Following whole-mount *in situ* hybridization, embryos were equilibrated in graded sucrose (15% and 30%) at 4°C, incubated overnight in 20% gelatine in 30% sucrose at 50°C and embedded in 20% gelatin at 50°C. The blocks were frozen on dry ice, mounted in TissueTek OCT and cryosectioned at a thickness of 35 µm.
